# Thoracic disc herniations: diagnosis, surgical techniques, and complication insights

**DOI:** 10.55730/1300-0144.5939

**Published:** 2024-12-30

**Authors:** Mehmet Denizhan YURTLUK, Aydın Sinan APAYDIN, Hakan KINA, Khoi D. THAN

**Affiliations:** 1 School of Medicine, Bezmialem Vakıf University, İstanbul, Turkiye; 2 Department of Neurosurgery, Karabük University, Karabük, Turkiye; 3 Department of Neurosurgery, İstinye University, İstanbul, Turkiye; 4 Department of Neurosurgery, Duke University, Durham, NC, USA

**Keywords:** Thoracic disc herniations, myelopathy, surgical approaches, spine surgery

## Abstract

Thoracic disc herniations (TDHs) are rare conditions, comprising 0.1% to 5% of all reported herniation cases, and affecting up to 1 in 1,000,000 individuals. The mid to lower thoracic spine, particularly between the T11 and T12 vertebrae, is most frequently affected and often associated with trauma. Conditions like Scheuermann’s disease can predispose patients to TDHs. This study explores the clinical manifestations, diagnostic methods, surgical techniques, and complications associated with TDHs. Diagnosis relies on magnetic resonance imaging and computed tomography myelography to identify the level and nature of the herniation. Various surgical approaches, including posterolateral, lateral, and anterior, have been implemented, each with specific indications, advantages, and pitfalls. Complications range from lung-associated to neurological deterioration to dural breaches. Clinical presentation primarily includes thoracic back pain. Patients can present with significant neurological deficits depending on the herniation’s characteristics, namely giant centrally located. Surgical intervention is indicated in cases of failed conservative treatment or acute trauma with significant cord compression. This review aimed to delve into the literature to provide insights into the clinical manifestations of TDHs, diagnosis, surgical techniques, and associated complications.

## 1. Introduction

Thoracic disc herniations (TDHs) are relatively rare conditions of the spinal column, comprising 0.1% to 5% of all reported herniation cases and affecting 1 in 1000 to 1 in 1,000,000 people in the general population [1–3]. A history of trauma is usually present, and patients with Scheuermann’s disease are especially susceptible to developing TDHs [4,5]. TDHs generally occur in the mid-lower thoracic area of the spinal column or below the T7–T8 vertebra in 75% percent of cases, most commonly between T11 and T12 [5,6]. This is thought to be due to the greater mobility of the posterior longitudinal ligament (PLL) at T11–T12, making the disc at this level more prone to degeneration. Additionally, thoracic kyphotic curvature in this region is the distribution center of axial forces, increasing the strain on biomechanic components of the spinal column most particularly the discs and facet joints [5,7]. Moreover, segmental PLL ossification is found to be a factor rendering TDH symptomatic since the spinal canal is wider at the distal thoracic region, and ossified PLL can act as calcified disc prolapse [8,9]. Thoracic back pain is the most common presenting symptom in 92% of cases and can also present as cervical or lumbar pain, whether localized above T5 or below T10, respectively [5]. Persistent headaches due to meningeal irritation or Horner syndrome due to T1 nerve root compression can rarely be seen [10]. Several surgical approaches have been developed to treat TDHs, considering their size, calcification, and dural extension, and address the shortcomings of other approaches. The current review aimed to assess the clinical and radiological presentations of TDHs and surgical approaches with their shortcomings and associated complications.

## 2. Methods

The literature was reviewed using specific keywords and boolean operators to identify relevant articles. Pubmed, ScienceDirect, Scopus, and Web of Science were searched using the following search queries: (thoracic disc herniation or thoracic intervertebral disc herniation), and (clinical diagnosis or diagnosis, or imaging, or clinical presentation), and (surgical techniques or surgery, or operative management, or minimally invasive surgery, or endoscopic surgery), and (management of complications or complications, or postoperative complications, or outcomes). Relevant articles were screened for diagnosis, management, and complications for narrative review synthesis, and any non-English studies were excluded from the review.

## 3. Clinical presentation and radiological diagnosis

As mentioned above, the most common presenting symptom is thoracic back pain, but TDH can generate myelopathy signs due to cord compression as well. Some patients can present with ataxia and motor deficits in the lower extremities due to compression of the spinothalamic tract and corticospinal tracts, respectively. Moreover, patients can present with bladder symptoms or lesional or sublesional symptoms, including intercostal or abdominal pain, respectively [5]. Quint et al. [2] found that 11% of cases are sudden-onset posttraumatic deficits, including paraparesis, BrownSequard syndrome, Vesical-sphincter disorders, and paraplegia. Several factors render the spinal cord susceptible to injury by compression. Primarily, intradural extension of central giant calcified herniations can compress the spinal cord. Second, the thoracic kyphosis forces the dural sheet to lie flat against the posterior spinal cord, and the dentate ligament within the dural sheath limits the mobility of the spinal cord, adding to the risk of compression. Furthermore, the diameter of the spinal cord (6.5 × 8 mm) is large relative to the spinal canal diameter (16.8 × 17.2 mm) in this region, leaving less free space around the spinal cord. Finally, watershed zones in the thoracic spinal cord due to poor vascularization make them prone to ischemia with compression [5,11]. For instance, a few rare cases have presented as permanent paraplegia due to occlusion of the anterior spinal artery by a central hernia [12].

Plain radiographs may be useful in cases of calcified protrusion, but in most cases, magnetic resonance imaging (MRI) and computed tomography (CT) myelogram are the two most important imaging tools to determine the vertebral level (Figure). Furthermore, a whole spinal image should be obtained to rule out any concomitant cervical or lumbar herniation/stenosis. Thoracic CT scan is important to determine the nature of the disc (calcified or noncalcified) and the localization within the spinal canal, which can be further divided into central, posterolateral, lateral, and far lateral [5]. An intramedullary low signal on T1 sequences or hyperintensity on T2 sequences are the best indicators for myelopathy [5]. Following the failure of conservative management and during the preoperative planning, it is vital to locate the dominant thoracolumbar artery (Adamkiewicz’s artery), which is the major anterior medullary blood supply for the spinal cord. Locating this artery is vital for anterior transthoracic approaches such as anterior thoracotomy or thoracoscopy. Therefore, obtaining an arteriography preoperatively is crucial. However, this procedure is invasive and can be overcome by replacing it with an MRI angiography, although with decreased resolution [13]. For anatomical purposes, Adamkiewicz’s artery usually resides on the left posterior thoracic wall arising between T9–L1 [5].

## 4. Surgical indications and techniques

### 4.1. Making a clinical diagnosis and preoperative planning

In simple cases with isolated back pain without any previous neurologic deficits, patients usually respond well to conservative management. Surgery is generally indicated following the failure of conservative management with persistent or worsening symptoms. However, in the case of acute trauma with severe cord compression, immediate surgery is warranted. Patients with myelopathy signs on MRI may benefit from surgical treatment even in the absence of significant clinical signs [5].

Marking the true level of herniation is vital for preoperative planning and to decrease the risk of postoperative complications. Several techniques have been described to mark the exact level of herniation preoperatively to aid in surgical planning. Placing adhesive radiographical markers on sagittal MRI, percutaneous methylene blue dye injection into tissues surrounding spinous processes, vertebroplasty with polymethylmethacrylate in cases of vertebral fractures, coil deployment to the periosteum at the appropriate level, and flexible hook-wire or K-wire placement into various anatomical landmarks under image guidance [14–19]. Moreover, the placement of a subpleural metal harpoon on the superior margin of the rib at the herniated level or coil injection to the intercostal artery at the level of herniation during preoperative arteriography has been described as an additional technique [5,11]. Unfortunately, the data on these techniques are scarce, and these are invasive procedures that may carry additional complication risks. The use of intraoperative CT scans can alleviate the need for preoperative invasive marking and improve surgical outcomes [5].

### 4.2. Surgical approaches

Several surgical techniques have been developed to decrease postoperative mortality and morbidity and to overcome the shortcomings of previous techniques (Table 1). Initially, posterior laminectomy with thoracic discectomy was developed, but due to high rates of postoperative neurologic deficits, it was abandoned over time, and several other approaches have been developed, including posterolateral, lateral, and anterior. Unfortunately, none of these approaches has been selected as the gold standard, and the choice of the technique depends on the properties of the herniated disc, namely the calcification status and location within the spinal canal.

### 4.3. Posterolateral approach

The posterolateral (PL) approach has several variations, namely pedicular-transfacet and transfacet variation, which spare the pedicle [11]. Regardless of the variation, the main principle of the approach remains the same. The PL approach is indicated for noncalcified soft herniations that are either in posterolateral or lateral trajectory or in cases of compression due to ossification of the PLL in multiple subsequent levels [20,21]. A straight skin incision is made following positioning of the patient in the prone position. Lamina and transverse process are exposed using blunt dissection and unipolar cautery for bleeding control. For extensive hernias, pedicles can be burred for better access or otherwise can be preserved [22,23]. A hemilaminectomy can be made to visualize the lateral aspect of the spinal cord [5]. Furthermore, some researchers have suggested the use of an angled endoscope to reach the anterior portion of the dura without any damage to the dura itself [24]. A calcified disc poses a challenge while separating the disc from the dura and may cause the rupture of the dura and facilitate dural-pleural fistula formation. To avoid this, the calcified outer portion of this disc can be left on the dura [21]. Moreover, ligation of the corresponding nerve root at that level can improve the surgical field while separating the disc from the anterior portion of the dura [11]. Several researchers have described transpedicular microdiscectomy through endoscope tubes or endoscopic discectomy techniques in several reports with considerable patient satisfaction and decreased postoperative complications, including less operative bleeding, less postoperative pain, and less instability [20,25–27]. Posterior fusion at the operated level can be made in cases of arthrectomy reaching into the pedicle; however, preservation of the lateral aspect of the articular facet can alleviate the need for posterior fusion by not destabilizing the structure of the spinal column. Naturally, the posterolateral approach does have several shortcomings. To name a few: It is recommended for soft, noncalcified, lateral herniations and does not offer adequate visualization for centrally located herniations. Furthermore, instrumentation is needed in a significant portion of the cases due to disruption of anatomical articular landmarks, increasing instability.

### 4.4. Lateral approach

The lateral approach provides more lateral access to disc space, going through retropleural space. The lateral extra cavitary approach first started to be used in 1976, offering better central visualization than the PL approach and without the pulmonary drawbacks of the anterior approaches, namely the pleural injury and atelectasis. It also provides the chance of posterior instrumentation from the same incision [11]. The patient is positioned in the prone position, and a midline curvilinear incision is made, blunt dissection is made laterally to identify the facet joint and the rib head at the intended level. The visualized rib head is from the costa one level below the pathological level (e.g., T10–T11pathology, the rib head is from the T10 costa). A vertical incision is made parallel to the dorsal surface of the dorsal wall of the rib cage to mobilize the latissimus dorsi for resection of the facet complex, transverse process, and proximal rib head. Additionally, the neurovascular bundle can be ligated as well to achieve better visualization of the lateral and anterior aspects of the spinal cord. Furthermore, hemilaminectomy again increases exposure to the lateral and dorsal aspects of the thecal sac [11]. The surgeon accomplishes vertebrectomy through the drilling of the disc space both above and below, then pushes the calcified disc into the artificial cavity, accomplishing the decompression. This technique was later abandoned due to excessive soft tissue dissection and damage.

### 4.5. Anterior approach

Anterior approaches provide unprecedented access to centrally located hernias. The anterior approach is initially done with thoracotomy, which requires intubation of the patient and deflation of the lung at the surgical site. This technique carries potential risks for morbidity and has led to the development of thoracoscopy. However, thoracoscopy has the disadvantage of using a camera as the source of magnification, which is very limited compared to a surgical microscope. Moreover, thoracoscopy has a lengthy learning curve [11]. Open minithoracotomy was later developed to overcome the shortcomings of previous anterior techniques. Several researchers have stated that mini open-thoracotomy is the best approach for centrally located calcified TDHs [28,29]. Protection of the pleura and the anterior portion of the dura, especially in calcified herniations with intradural extension, is vital. Mini-open thoracotomy does not require transpleural access to disc space, therefore reducing lung-related complications [30]. The anterior approach also involves resecting the rib head to access the anterior dural surface. Conical clearing of the posterior surface of the disc creates free space for the remaining disc and hernia to be pushed back from the medullary cavity and decompress the spinal cord anteriorly [11].

## 5. Discussion

### 5.1. Complications associated with surgery and how to address them

According to a recent meta-analysis by Brotis et al. [31], the morbidity associated with TDH discectomy and decompressions varied between 12% and 48%. They revealed that the posterolateral approach offered decreased rates of morbidity when compared to anterior and lateral approachesClick or tap here to enter text.. Their analysis also showed that the anterior approach was associated with a higher likelihood of postoperative morbidity, medical complications (e.g., stroke, pneumonia, pulmonary embolism, seizure, and deep vein thrombosis), cerebral spinal fluid-related complications, and local nerve irritation, which resulted in intercostal neuralgiaClick or tap here to enter text.. The complications can be classified as lung-associated, neurological deterioration, and dural breaches.

### 5.2. Lung-associated complications

Lung-associated complications are mainly seen in anterior approaches. The transpleural approach is associated with atelectasis, pleural effusion, or pneumopathy [11]. Although reversible, they increase the postoperative hospital stay for patients and, unfortunately, fail to decrease the incidence of lung-associated complications when compared to mini-thoracotomy [11].

### 5.3. Neurologic deterioration

Watershed areas within the medulla of the cord make it susceptible to ischemic injury during the operation. Cases can range from monoplegia to paraplegia. These cases are generally reversible, but they have the potential to increase morbidity in patients with comorbidities. Court et al. [11] made several suggestions to prevent injury to the spinal cord, including 1) adequate exposure of the dural sheet; 2) using a microscope, as it provides excellent visualization of the surgical field; 3) maintaining a mean arterial blood pressure of more than 80 mmHg peri and postoperatively; and 4) preoperative administration of high doses of corticosteroids [32]. Added to this can be avoiding excessive manipulation of the spinal cord and using intraoperative neurophysiologic monitoring. There is no consensus on using intraoperative monitoring, but a decrease in the amplitude by a 50% cutoff value has a high sensitivity and specificity [33].

### 5.4. Dural breaches

Dural breaches most commonly occur during the resection of giant calcified herniations with intradural extension. Gille et al. [34] reported that 39% of patients had cerebrospinal fluid (CSF) leaks due to dural breaches. They recommended leaving a portion of the calcified shell on the dural surface to avoid dural tears. Patients can develop dyspnea, thoracic pain, tachypnea, and pleural effusion if the dural tear is overlooked and not treated appropriately [5]. Over time, a pleural-dural fistula can form and present with neurological signs, including headache, vertigo, nausea, and diplopia, weeks or months after the surgery. The pathophysiology of the fistula formation is thought to be aspiration due to negative intrapleural pressure (−2 and −8 cm H_2_O). Moreover, brain imaging demonstrates a reduction in the ventricular size [11]. Surgical revision is required for fistula treatment in 50% of cases [35]. Fibrin glue application, continuous lumbar draining, and repeated lumbar puncture (spinal tap) can also be used for fistula treatment. Court et al. [11] also treated two patients with percutaneous coil injection into the disc space to seal the dural fistula. CT myelogram must be obtained for correct localization of the fistula. In the case of intraoperative detection of the breach, immediate closure with sutures must be implemented, followed by muscle or aponeurosis tissue to support the closure [11]. Continuous lumbar drainage and maintaining ventilation with peak positive end-expiratory pressure during the intubated period and continuous positive airway pressure following extubation are vital to avoid CSF leak-associated complications [11].

## 6. Conclusion

TDHs present significant challenges in terms of finding the appropriate surgical approach and managing complications. Choosing the right approach depends on the characteristics of the herniation, with each surgical technique having distinct advantages and pitfalls. Surely, intraoperative navigation systems will play a crucial role in improving patient outcomes and decreasing the risk of generating surgical complications.

## Figures and Tables

**Figure:**
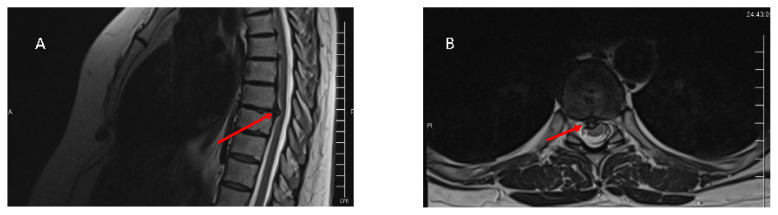
A 51-year-old female patient who presented with several months of back pain. Further investigation revealed a TDH at the level of T9–10. **A.** Coronal T2 image demonstrating TDH (red arrow) compressing the spinal cord at the same level. **B.** Axial T2 image demonstrating the same TDH (red arrow) compressing the spinal cord at the same level. Fortunately, there were no signs of myelopathy on the MRI scans or signs of radiculopathy at the patient’s presentation.

**Table:** **Studies** reporting the results of different a**pproaches.**

Author	No.	Mean age	Female/male	Surgical approach	Most common thorcic level	Most common presenting symptoms	Mean follow-up	Complications
**Arts et al.** [**20**]	100	52.4/56.9	33/23, 26/18	Mini-TTA/P, 56/44	T9–10/T11–12	Sensory deficit/sensory deficit	4.1 years	Mini-TTA: 10 CSF leakage, Pneumonial, Pleaural Effusion, Neurologic Deterioration: 2 Mini-TTA, P. 1 Wrong Level Surgery
**Carr et al.** [**22**]	51	60	31/20	PL	T11–12	Radiculopathy	14 months	1 Postoperative epidural hematoma, 3 suboptimal hardware placement, 1 migrated bone fragment, 1 anterior migration of interbody cage into mediastinum, 1 long pedicle screw associated hemorrhage, 1 pseudoarthrosis, 2 postoperative wound infections
**Nie et al.** [**25**]	13	51.4	6/7	Endoscopic PL	T7–8	Back pain	17 months	1 patient spinal positional headache, 1 Recurrent TDH
**Russo et al.** [**28**]	7	53	4/3	Anterior mini-thoracotomy	2 T5–6, 2 T7–8, 2 T9–10	Neurologic deficits	23.5 months	1 Patient Abdusence Palsy, 2 Intercostal Neuralgia, 4 CSF Leakage
**Bartels et al.** [**29**]	28	52.8/54.8	12/9, 5/2	Mini-thoracotomy/thoracoscopy, 21/7	T11–12/T9–10	Gait disturbance, myelopathic changes	NA	Mini-thoracotomy: 2 CSF leakage thoracoscopy: 2 neuropathic pain
**Moran et al.** [**30**]	17	50 (median)	9/8	Mini-open retropleural transthoracic	T8–9	Myelopathy, motor signs	NA	5 Pleural tears, 1 CSF leakage, 1 pulmonary embolism, 1 patient died due to pnumococcus pneumonia
**Cornips et al.** [**33**]	77	49	52/25	Anterior-transthoracis	T9–10	Myelopathy	NA	1 Paralysis, 1 patient died due to infected pleural hematoma, 2 CSF leakage, 2 pleural hematomas.

**Mini-TTA**: mini-transthoracic approach, **P**: posterior, **CSF**: cerebrospinal fluid **NA**: not available.

## References

[1] Stillerman CB Chen TC Couldwell WT Zhang W Weiss MH Experience in the surgical management of 82 symptomatic herniated thoracic discs and review of the literature Journal of Neurosurgery 1998 88 4 623 633 10.3171/jns.1998.88.4.0623 9525706

[2] Quint U Bordon G Preissl I Sanner C Rosenthal D Thoracoscopic treatment for single level symptomatic thoracic disc herniation: a prospective followed cohort study in a group of 167 consecutive cases European Spine Journal 2012 21 4 637 645 10.1007/s00586-011-2103-0 22160099 PMC3326138

[3] Hott JS Feiz-Erfan I Kenny K Dickman CA Surgical management of giant herniated thoracic discs: analysis of 20 cases. Journal of Neurosurgery Spine 2005 3 3 191 197 10.3171/spi.2005.3.3.0191 16235701

[4] Palazzo C Sailhan F Revel M Scheuermann’s disease: an update Joint Bone Spine 2014 81 3 209 214 10.1016/j.jbspin.2013.11.012 24468666

[5] Bouthors C Benzakour A Court C Surgical treatment of thoracic disc herniation: an overview International Orthopaedics 2019 43 4 807 816 10.1007/s00264-018-4224-0 30406842

[6] Arce CA Dohrmann GJ Herniated thoracic disks Neurologic Clinics 1985 3 2 383 392 10.1016/S0733-8619(18)31043-0 3894922

[7] Dietze DD Fessler RG Thoracic disc herniations Neurosurgery Clinics of North America 1993 4 1 75 90 10.1016/S1042-3680(18)30609-0 8428158

[8] He B Yan L Xu Z Guo H Liu T Treatment strategies for the surgical complications of thoracic spinal stenosis: a retrospective analysis of two hundred and eighty three cases International Orthopaedics 2014 38 1 117 122 10.1007/s00264-013-2103-2 24057658 PMC3890145

[9] Moon SJ Lee JK Jang JW Hur H Lee JH The transdural approach for thoracic disc herniations: a technical note European Spine Journal 2010 19 7 1206 1211 10.1007/s00586-010-1294-0 20143105 PMC2900008

[10] Morgan H Abood Disc herniation at T1–2. Report of four cases and literature review Journal of Neurosurgery 1998 88 1 148 150 10.3171/jns.1998.88.1.0148 9420090

[11] Court C Mansour E Bouthors C Thoracic disc herniation: surgical treatment Orthopaedics and Traumatology Surgery and Research 2018 104 1S S31 S40 10.1016/j.otsr.2017.04.022 29225115

[12] Guest JD Griesdale DE Marotta T Thoracic disc herniation presenting with transient anterior spinal artery syndrome: a case report Interventional Neuroradiology 2000 6 4 327 331 10.1177/159101990000600408 20667212 PMC3679707

[13] Takagi H Ota H Natsuaki Y Komori Y Ito K Identifying the Adamkiewicz artery using 3-T time-resolved magnetic resonance angiography: its role in addition to multidetector computed tomography angiography Japanese Journal of Radiology 2015 33 12 749 756 10.1007/s11604-015-0490-6 26497024

[14] Rosahl SK Gharabaghi A Liebig T Feste CD Tatagiba M Skin markers for surgical planning for intradural lesions of the thoracic spine: technical note Surgical Neurology 2002 58 5 346 348 10.1016/s0090-3019(02)00863-7 12504308

[15] Paolini S Ciappetta P Missori P Raco A Delfini R Spinous process marking: a reliable method for preoperative surface localization of intradural lesions of the high thoracic spine British Journal of Neurosurgery 2005 19 1 74 76 10.1080/02688690500089209 16147592

[16] Hsu W Sciubba DM Sasson AD Khavkin Y Wolinsky JP Intraoperative localization of thoracic spine level with preoperative percutaneous placement of intravertebral polymethylmethacrylate Journal of Spinal Disorders and Techniques 2008 21 1 72 75 10.1097/BSD.0b013e3181493194 18418141

[17] Binning MJ Schmidt MH Percutaneous placement of radiopaque markers at the pedicle of interest for preoperative localization of thoracic spine level Spine 2010 35 19 1821 1825 10.1097/BRS.0b013e3181c90bdf 20543770

[18] Sammon PM Gibson R Fouyas I Hughes MA Intra-operative localisation of spinal level using pre-operative CT-guided placement of a flexible hook-wire marker British Journal of Neurosurgery 2011 25 6 778 779 10.3109/02688697.2011.584987 21707263

[19] Thambiraj S Quraishi NA Intra-operative localisation of thoracic spine level: a simple ‘‘K’-wire in pedicle’ technique European Spine Journal 2012 21 Suppl 2 221 224 10.1007/s00586-012-2193-3 PMC3326084 22349971

[20] Arts MP Bartels RHMA Anterior or posterior approach of thoracic disc herniation? A comparative cohort of mini-transthoracic versus transpedicular discectomies The Spine Journal 2014 14 8 1654 1662 10.1016/j.spinee.2013.09.053 24374099

[21] Kato S Murakami H Demura S Yoshioka Y Hayashi H Gradual spinal cord decompression through migration of floated plaques after anterior decompression via a posterolateral approach for OPLL in the thoracic spine Journal of Neurosurgery Spine 2015 23 4 479 483 10.3171/2015.1.SPINE14960 26140403

[22] Carr DA Volkov AA Rhoiney DL Setty P Barret RJ Management of thoracic disc herniations via posterior unilateral modified transfacet pedicle-sparing decompression with segmental instrumentation and interbody fusion Global Spine Journal 2017 7 6 506 513 10.1177/2192568217694140 28894679 PMC5582705

[23] Stillerman CB Chen TC Day JD Couldwell WT Weiss MH The transfacet pedicle-sparing approach for thoracic disc removal: cadaveric morphometric analysis and preliminary clinical experience Journal of Neurosurgery 1995 83 6 971 976 10.3171/jns.1995.83.6.0971 7490640

[24] Paolini S Tola S Missori P Esposito V Cantore G Endoscope-assisted resection of calcified thoracic disc herniations European Spine Journal 2016 25 1 200 206 10.1007/s00586-015-3858-5 25761864

[25] Nie HF Liu KX Endoscopic transforaminal thoracic foraminotomy and discectomy for the treatment of thoracic disc herniation Minimally Invasive Surgery 2013 2013 264105 10.1155/2013/264105 24455232 PMC3880763

[26] Chi JH Dhall SS Kanter AS Mummaneni PV The mini-open transpedicular thoracic discectomy: surgical technique and assessment Neurosurgcial Focus 2008 25 2 E5 10.3171/FOC/2008/25/8/E5 18673053

[27] Xiaobing Z Xingchen L Honggang Z Xiaoqiang C Qiadong Y ‘U’ route transforaminal percutaneous endoscopic thoracic discectomy as a new treatment for thoracic spinal stenosis International Orthopaedics 2019 43 4 825 832 10.1007/s00264-018-4145-y 30218183

[28] Russo A Balamurali G Nowicki R Boszczyk BM Anterior thoracic foraminotomy through mini-thoracotomy for the treatment of giant thoracic disc herniations European Spine Journal 2012 21 Suppl 2 212 220 10.1007/s00586-012-2263-6 PMC3326080 22430542

[29] Bartels RHMA Peul WC Mini-thoracotomy or thoracoscopic treatment for medially located thoracic herniated disc? Spine (Phila Pa 1976) 2007 32 20 581 584 10.1097/BRS.0b013e31814b84e1 17873799

[30] Moran C Ali Z McEvoy L Bolger C Mini-open retropleural transthoracic approach for the treatment of giant thoracic disc herniation Spine 2012 37 17 E1079 E1084 10.1097/BRS.0b013e3182574657 22475729

[31] Brotis AG Tasiou A Paterakis K Tzerefos C Fountas KN Complications associated with surgery for thoracic disc herniation: a systematic review and network meta-analysis World Neurosurgery 2019 132 334 342 10.1016/j.wneu.2019.08.202 31493617

[32] Strom RG Mathur V Givans H Kondziolka DS Perin NI Technical modifications and decision-making to reduce morbidity in thoracic disc surgery: an institutional experience and treatment algorithm Clinical Neurology and Neurosurgery 2015 133 75 82 10.1016/j.clineuro.2015.03.014 25867235

[33] Cornips E Habets J van Kranen-Mastenbroek V Bos H Bergs P Anterior Transthoracic Surgery with Motor Evoked Potential Monitoring for High-Risk Thoracic Disc Herniations: Technique and Results World Neurosurgery 2017 105 441 455 10.1016/j.wneu.2017.05.173 28599909

[34] Gille O Soderlund C Razafimahandri HJC Mangione P Vital JM Analysis of hard thoracic herniated discs: review of 18 cases operated by thoracoscopy European Spine Journal 2006 15 5 537 542 10.1007/s00586-005-1014-3 16408236 PMC3489338

[35] Ohana N Benharroch D Sheinis D Pleural dural fistula after anterior excision of thoracic disc hernia: suggested repair procedure The Annals of Thoracic Surgery 2019 107 3 e191 e193 10.1016/j.athoracsur.2018.07.065 30266610

